# Adverse Features of Rectourethral Fistula Requiring Extirpative Surgery and Permanent Dual Diversion: Our Experience and Recommendations

**DOI:** 10.3390/jcm10174014

**Published:** 2021-09-05

**Authors:** Francisco E. Martins, João Felicio, Tiago Ribeiro Oliveira, Natália Martins, Vítor Oliveira, Artur Palmas

**Affiliations:** 1 Department of Urology, School of Medicine, University of Lisbon, Hospital Santa Maria (CHLN), 1649-028 Lisbon, Portugal; jpfelicio@gmail.com (J.F.); tiagoribeirooliveira@sapo.pt (T.R.O.); 2 Hospital das Forças Armadas, 1649-020 Lisbon, Portugal; natalia.mashanova.martins@gmail.com (N.M.); palmas.artur@gmail.com (A.P.); 3 Department of Urology, Centro Hospitalar Vila Nova de Gaia (CHVNG), 4434-502 Porto, Portugal; vitorolive@gmail.com

**Keywords:** urinary fistula, pelvic cancer, prostate cancer, radiotherapy, reconstructive surgery

## Abstract

Introduction: To report a series of men with a rectourethral fistula (RUF) resulting from pelvic cancer treatments and explore their therapeutic differences and impact on the functional outcomes and quality of life highlighting the adverse features that should determine permanent urinary or dual diversion. Methods: A retrospective database search was performed in four centers to identify patients with RUF resulting from pelvic cancer treatment. Medical records were analyzed for the demographics, comorbidities, diagnostic evaluation, fistula characteristics, surgical approaches and outcomes. The endpoints analyzed included a successful fistula closure following a repair and the impact of the potential adverse features on outcomes. Results: Twenty-three patients, aged 57–79 years (median 68), underwent an RUF reconstruction. The median follow-up (FU) was 54 months (range 18–115). The patients were divided into two groups according to the etiology: radiation/energy-ablation treatments with or without surgery (G1, *n* = 10) and surgery only (G2, *n* = 13). All of the patients underwent a temporary diverting colostomy and suprapubic cystostomy. Overall, a successful RUF closure was achieved in 18 (78%) patients. An interposition flap was used in six (60%) patients and one (7.7%) patient in groups G1 and G2, respectively (*p* = 0.019). The RUF was managed successfully in all 13 patients in group G2 as opposed to 5/10 (50%) in group G1 (*p* = 0.008). The patients in the radiation/energy-ablation group were more likely to require permanent dual diversion (50% vs. 0%, *p* < 0.0075). Conclusion: Radiation/energy-ablation therapies are associated with a more severe RUF and more complex reconstructions. Most of these patients require an abdominoperineal approach and flap interposition. The failure of an RUF repair with the need for permanent dual diversion, eventually combined with extirpative surgery, is higher after previous radiation/energy-ablation treatment. Therefore, permanent dual diversion as the primary treatment should always be included in the decision-making process as reconstruction may be futile in specific settings.

## 1. Introduction

A rectourethral fistula (RUF) is a complication of pelvic cancer treatment, with an incidence of 0.4–3% [1]. It is associated with a debilitating morbidity and a significant impact on the quality of life (QoL) [2]. Radiation is an effective and essential therapeutic modality in pelvic oncology. However, over the last decades, the widespread use of radiation and other energy-ablation therapies for pelvic malignancies has led to an increase of complex iatrogenic fistulae that are seen by urological and colorectal surgeons. Most RUF are iatrogenic, either from surgery alone or from non-surgical, energy-ablation treatment modalities, or a combination of these [1].

Conservative treatment is rarely successful, except in small, non-radiated fistulae. Therefore, most patients will require surgery, and multiple surgical techniques have been proposed with variable success rates [3,4]. Several studies have retrospectively assessed the impact of radiotherapy and energy-ablation treatments on patients’ reconstructive outcomes [5,6,7,8,9]. These treatment modalities induce significant fibrosis and vascular damage. Because of the heterogeneity of the RUF characteristics after radiation/energy-ablation, ranging from minimal changes in surrounding tissue to extensive local damage, there is no standardized approach for its treatment.

The combination of specific adverse features that induce severe damage to local surrounding tissues and the complexity of the surgical reconstruction, in the presence of these adverse features, significantly increase the potential for a fistula recurrence after the primary management.

We report a series of men with RUF resulting from pelvic cancer treatments and explore the differences and impact on outcomes between these treatments and the presence of any adverse features that required permanent urinary and, with or without concomitant, fecal diversion.

## 2. Methods

The medical records were retrospectively analyzed for 23 consecutive patients with RUF after treatment for pelvic malignancies who underwent surgical reconstruction in our centers between January 2008 and December 2018. All of the patients had at least an 18-month follow-up (FU) since their last intervention. Due to differences in treatment, outcomes and impact on the QoL, we divided patients into two major groups: (1) radiotherapy/energy-ablation ± surgery (G1); (2) surgery alone (G2).

The medical records were reviewed including demographic data, symptoms, comorbidities, fistula characteristics (e.g., fistula size, health of adjacent tissues, pelvic necrotic cavity, infection, abscess, exposure to radiation or energy-ablation therapy), type of fistula repair, bladder and bowel function, pretreatment continence status, follow-up and outcomes. All of the patients underwent a digital rectal examination and flexible cystourethroscopy, retrograde urethrography/voiding cystourethrography (RUG/VCUG), a pelvic computed tomography (CT) scan with 3D reconstruction and magnetic resonance imaging (MRI) in the routine preoperative assessment. Three patients following an anterior rectal resection received a barium enema with a fistulogram and rectosigmoidoscopy requested by their referring colorectal surgeons. 

The primary endpoint of reconstruction success was defined as spontaneous urethral voiding without leakage from the rectum and avoidance of any permanent diversion. Urinary incontinence after an RUF closure was not considered a fistula reconstructive failure. All of the patients were previously informed about the possibility of further treatment for urinary incontinence. All of the surgeries were performed by the same senior surgeon (FEM).

## 3. Statistical Analysis

All of the data is reported in absolute and relative frequencies for the categorical variables and in medians for the continuous variables. A Student’s *t*-test was used for the significance between the two groups’ medians age, body mass index, serum albumin levels, smoking habits, diabetes mellitus (DM) and hypertension. The Fisher’s exact test was used to assess the significance of overall success, number of surgical attempts required and the need of interposition tissue for success in the two patient cohorts. A value of *p* < 0.05 was considered statistically significant. The statistical analysis was performed with the SPSS Software (Version 19, IBM, Chicago, IL, USA).

## 4. Results

**Figure 1:**
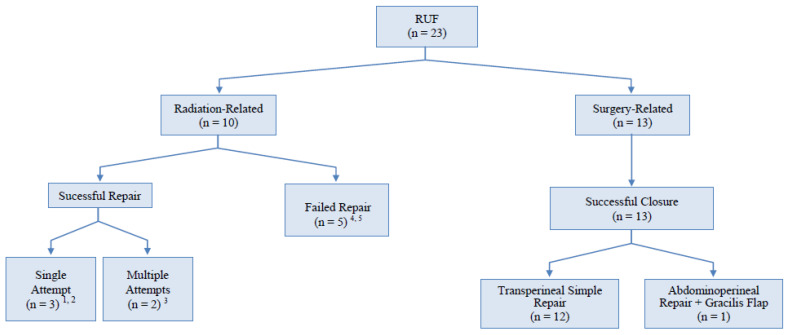
Algorithm of surgical management of RUF followed in our study.^1^ One transperineal repair + gracilis; ^2^ two abdominoperineal repairs (one gracilis; one omentum); ^3^ two abdominoperineal repairs (both omentum); ^4^ three transperineal repairs (one Gracilis; two none); ^5^ two abdominoperineal repairs (one none; one glubran).

The patients’ median age at the time of the reconstruction was 68 years (range 57–79). If grouped according to etiology, the median ages for groups G1 and G2 were 66.1 and 69.15, respectively (*p* = 0.279). A FU was available for all of the patients and ranged from 18 to 115 months (median 54). Ten (43%) patients were classified according to the etiology to G1 and 13 (57%) to G2. The radiation dosimetric parameters to the bladder neck and posterior urethra ranged from 60 Gy to 155 Gy if adjuvant external beam radiotherapy (EBRT) or combined EBRT + brachytherapy (BT) were used, respectively. The clinical characteristics of the patients, comorbidities, surgical outcomes and clinical implications of pelvic cancer treatments by patient cohort are summarized in Table 1, Table 2 and Table 3, and in Figure 1, respectively. There were no differences among the two groups regarding smoking habits (20% vs. 30.8%, *p* = 0.199) and nutritional status (serum albumin, 3.81 vs. 3.60, *p* = 0.284) (Table 2.) All of the patients presented with urine leakage from the rectum, with fecaluria present in five (22%) patients. Three patients after radiotherapy (one of them with salvage high-intensity focused ultrasound (HIFU)) presented with rectal/pelvic pain and rectal bleeding. No fistula closed spontaneously in our series.

Two surgical approaches were used: transperineal in 16/23 (70%) patients and abdominoperineal in 7/23 (30%) based on the local tissue integrity, fistula characteristics, previous radiation/energy-ablation exposure, bowel and bladder function including the status of the urethra and preoperative continence and the presence of pelvic sepsis. Of a total of 23 patients, 7 (30%) underwent a surgical repair with an interposition flap, 6 in G1 and 1 in G2 (*p* = 0.019). Three patients following radical prostatectomy (RP; open and laparoscopic) required vesicourethral re-anastomosis.

Overall, the successful closure of the fistula was achieved in a total of 18/23 (78%) patients, 13/13 (100%) in G2 and 5/10 (50%) in the G1 cohorts (*p* = 0.008). Of the 5/10 (50%) successful G1 patients, the fistula closure was achieved after one attempt in three patients, after two attempts (York-Mason followed by transabdominal) in one patient and after three attempts (one York-Mason and two transabdominal) in one patient, whereas all 13/13 (100%) of the G2 patients required one attempt only (*p* = 0.0005). A cystoprostatectomy was required in three G1 patients. One patient, after an anterior rectal resection, underwent a resection of his residual rectum for a pelvic infection and concomitant local tumor recurrence, maintaining his colostomy and opting for a suprapubic tube, despite a previously successful RUF closure. Synthetic biological glue (Glubran®) was used to aid the fistula closure in one patient following BT without success. However, both the permanent colostomy and urostomy were maintained in 5/10 (50%) patients, all in group G1 (50% vs 0%, *p* = 0.0075). No validated questionnaire was used in our study as we are not aware of any existing validated questionnaire in the literature and the main goal was to assess the clinical adverse features associated with an RUF reconstruction failure. Postoperative complications in the G1 cohort varied from Grade I (*n* = 4), requiring electrolyte repositioning, analgesics and antibiotics, to Grade II (*n* = 2), requiring a blood transfusion and Grade IIIA and IIIB (*n* = 3) according to the Clavien-Dindo classification [10]. Most of the patients in the G2 cohort developed Grade I–II (*n* = 4) and Grade IIIA and IIIB (*n* = 2) complications.

## 5. Discussion

Vesicourethral and other pelvic complications resulting from pelvic tumor treatments have increased in the last decades, and a new classification for a standardized report of complications has been proposed [10,11]. Although apparently less invasive, non-surgical treatments have been associated with more complex, incapacitating and harder-to-treat complications [2]. One of the major problems is that many clinicians have focused on oncologic therapeutic outcomes more than the impact of treatment on the QoL. Over the last three decades, considered a golden era to improve cancer survivorship, significant progress has been achieved to ensure optimal cancer management. However, the data on how best to the manage toxicity of radiation/energy-ablation is almost non-existent. The management of these patients should primarily reduce morbidity and improve the quality of life. Age did not impact surgical outcomes in our study. Overall, the success was significantly influenced by radiotherapy and the previous failed attempts, decreasing the likelihood of achieving a successful and lasting result without the need of permanent dual diversion.

An RUF represents one of the most challenging complications of pelvic radiotherapy, graded as “IV” according to the “Modified Radiation Therapy Oncology Group Lower Gastrointestinal Toxicity Scale” [12]. The occurrence rate after prostate brachytherapy ranges from 0.2% to 3% as a monotherapy, 2.9% in combined modality and 8.8% after salvage brachytherapy [12]. A review of 3834 radical prostatectomies found a mean incidence of rectal injuries of 0.7% (range 0.2–2.9%), regardless of the surgical approach [2].

A spontaneous RUF closure is possible through conservative measures in the absence of radiation or energy-ablation. However, there is no report that a postradiation RUF has closed definitively as spontaneous healing in this setting is exceedingly rare [2,13]. No RUF closed spontaneously in our series regardless of etiology.

A routine fecal diversion before or at the time of the fistula repair remains controversial. However, fecal diversion and a staged repair permit a trial of spontaneous healing of the fistula without open manipulation of the urinary tract. Nonetheless, a successful single-stage repair may spare the potential morbidity and cost of multiple staged repairs. The suggested guidelines for a one-stage approach include surgically induced, small, non-infected RUF with good bowel preparation. In contrast, staged repairs must be considered in large fistulae, an uncontrolled local or systemic infection, inadequate bowel preparation at the time of definitive repair and, importantly, in those associated with radiation/energy-ablative therapy [14]. We found it safer and more prudent to perform a colostomy in every patient. We could not avoid permanent dual diversion in five failed G1 patients and a colostomy in one additional G1 patient for an associated proctectomy despite the successful RUF closure. Combined brachytherapy and EBRT increase the risk of rectal toxicity with a significant impact on the patient’s QoL [12]. Noteworthy, severe rectal pain and/or ulceration is more common after radiation, making fecal diversion formally necessary. It is generally agreed that most radiation-induced RUF should undergo a permanent colostomy, and this has been reported in as many as 31% of cases [3,5]. All of the failed G1 patients opted for an ileal conduit to avoid further potential complications that might require surgical revision.

### 5.1. Surgical Approaches 

Currently, the main approaches to an RUF closure are the following: (1) transperineal; (2) transanal; and (3) abdominoperineal. Although we recognize the merits and efficacy in other techniques described in the literature, we believe the transperineal approach is a successful, single-stage method favored by many urologists for an RUF repair in most of the patients with significant advantages, including the possibility of flap interposition through the same incision. The transperineal approach also has the advantage of local access to a variety of potential interposition flaps with excellent results [15]. The three patients who failed the transperineal approach had received radiation, and HIFU in one in combination with radiotherapy (G1). In 16 (16/23; 70%) patients, one single surgical repair was enough for a successful RUF closure, with only three (3/16; 19%) of them in the G1 group. An abdominoperineal approach was used in seven (7/23; 30%) patients, deemed more complex patients (a combination of perineal cavity and radiation, multiple previous reconstructive attempts and the need of salvage extirpative procedures). Moreover, in most of these patients, salvage extirpative procedures, such as a radical prostatectomy/cystoprostatectomy and an omental flap, were required. All five failures in our study were in the G1 cohort and, although two underwent the abdominoperineal approach, the fistula could not be closed successfully. Four proceeded to receive extirpative surgery (a cystoprostatectomy in three and a total pelvic exenteration in one) with a permanent colostomy and ileal conduit urinary diversion. We believe an abdominoperineal approach should be considered and recommended in cases of difficult intraoperative access, significant surrounding tissue damage, mostly related to radiation or energy-ablation exposure or the need to perform concomitant extirpative pelvic surgery.

The York-Mason procedure appears to be effective and provides good exposure through unscarred tissue planes and, therefore, has long been used to treat an RUF after a radical prostatectomy [14]. Similar to others, however, we think that the York-Mason procedure is not advised in large and complex fistulae, and it should be strongly discouraged in patients with severe radiation proctopathy [2].

Some authors have used an alternative approach with success, specifically the transanal pull-through procedure with delayed coloanal anastomosis, in the treatment of lower rectal cancer with the preservation of anal integrity [16]. The authors found that this procedure had better results than the immediate coloanal anastomosis in terms of anastomotic leakage and major surgical complications. Furthermore, it did not require a temporary ileostomy.

### 5.2. Use of Tissue Interposition

The use of tissue interposition should be considered either to fill a defect when the remaining local tissue is unable to do so, or to bring the blood supply to support the complex anatomic reconstruction of tissues deprived of their native vascularization. Therefore, vascularized flaps are beneficial in radiation/energy-ablative settings. We do not recommend its routine and indiscriminate use in RUF caused exclusively by surgical injury, thus avoiding additional morbidity associated with these procedures [17]. We used a tissue flap interposition in a total of seven (7/23; 30%) patients, six of them after radiation. Tissue interposition was not used in other patients where a watertight closure could be achieved, including four radiated patients, which proved to be a mistake. Glubran® was used in one patient to support an apparently sealed closure and hypothetically improve watertightness. Unfortunately, this maneuver proved unsuccessful.

### 5.3. Impact of Radiation on Clinical Recovery, Pelvic/Rectal Pain, Urinary and Bowel Functions

In recent years, the number of patients submitted to multimodal treatment protocols for pelvic malignancies has risen and subsequently the incidence of RUF has increased [5,6,7]. Until 1997, only 3.8% of RUF involved pelvic radiation, rising to 52.6% from 1998 through 2012 [18,19]. We are witnessing, and dealing with, a surge of more complex RUF due to the increased use of combined energy-ablation treatments as salvage methods after failed primary therapies [20,21]. A salvage HIFU after the failure of radiation treatments for prostate cancer is associated with an increased rate of fistulation (3–6%) [22,23]. The high rate of RUF after this sequential treatment highlights how damaged and vulnerable the periprostatic area becomes after the combined treatment. In these instances, RUF are often large and necrotic, often associated with rectal pain and/or rectal ulceration, with poor tissue quality and impaired wound healing, preventing a safe and successful repair. The time interval between the exposure to radiation and diagnosis of an RUF has been reported to be up to 14 years [24]. Additionally, extreme caution should be exercised if a biopsy of the anterior rectum is deemed necessary in radiation proctopathy as these patients are increasingly vulnerable to fistulation [12,18,25]. Elective rectal surgery should also be precluded even several years after the completion of radiotherapy, which is not the case after surgery. Thus, we find it critical to discuss iatrogenic RUF in two different perspectives due to the therapeutic implications, success and prognosis depending on the presence of radiation/energy-ablation or surgery alone. Linder et al. reported that not only the primary repair was less frequently attempted and successful in radiated patients (87% vs. 17%) but also the primary radiotherapy was more likely to require a permanent colostomy (86% vs. 0%) and permanent urinary diversion (93% vs. 6%) as part of the RUF management [8]. Others reported an overall permanent fecal diversion rate of 25% in radiated vs. 3.8% in non-radiated patients [10]. Overall, the G2 (surgical) cohort had more favorable results, with all of our patients cured at the first surgical attempt and free of any form of diversion as opposed to 50% of the G1 patients requiring permanent diversion. Globally, the postoperative success rate was 78% (18/23), 100% success having been achieved in the G2 cohort (13/13) after a single attempt, compared to 50% in the G1 (5/10) after ≥ 1 attempt (Table 3). The five failures in the G1 cohort occurred after combined brachytherapy and EBRT, with HIFU in one patient. Failure can occur in these instances even with the interposition of a vascularized flap, as shown by some authors [2,4,12,26]. Four of them underwent pelvic extirpative surgery, maintaining both diversions permanently.

Recent advances have been made towards a better delivery of radiotherapy to reduce the rectal toxicity. The SpaceOAR™ System (Boston Scientific, Inc, Marlborough, MA, USA) is currently the only Food and Drug Administration-approved absorbable hydrogel spacer that can be introduced between the prostate and rectum to decrease the toxicity and minimize the changes in the QoL after prostate radiotherapy. Encouraging studies have shown evidence for its beneficial use in this setting [27,28].

Our lower success rate than reported in the literature in repairing radiation/energy-ablation fistulae is apparently because we have attempted to repair “high-risk” radiation fistulae that most urologists would wisely recommend for an exenteration and permanent diversion. We recognize that the adverse features that we address in our series should advocate against any reconstructive attempt, as we might be submitting patients to a wasted, futile surgery, and we should instead counsel permanent dual diversion in the initial decision-making process.

### 5.4. Study Limitations 

Our study has several weaknesses inherent to a retrospective and case-control study. The small sample size limits the interpretation and, although unintentionally, can introduce bias. Although these patients were treated in tertiary, teaching centers, we are aware of the steep learning curve and that better results can be achieved elsewhere.

## 6. Conclusions

Excellent results can be achieved for the primary repair of an RUF induced by surgery, even of a large size. Conversely, radiation/energy-ablation fistulae can be extremely difficult to successfully repair. Radiotherapy has a significant impact on the choice of surgical technique to treat the RUF. Patients who undergo multimodal therapies are at a higher risk of developing severe complications and repair failure, requiring more complex abdominoperineal operations with the interposition of vascularized tissue. Not uncommonly, patients with radiation/energy-ablation RUF will maintain dual diversion permanently, often after complex and repeated surgical, and eventually extirpative, procedures. The toxicity and quality of life following non-surgical treatments should be considered with extreme caution in individual patient counselling and treatment selection. Technological improvement is urgently needed to enable a safe and more precise delivery of radiation.

## Figures and Tables

**Table 1:** Patient clinical demographics, surgical characteristics and outcomes.

N°pts	Age (y)	Etiology	Location/Type of Fistula	N° Previous Attempts	Surgical Approach	Ureteric Stenting	Tissue Graft Interposition	Outcomes
Radiation/High Energy Ablation Group (*n* = 10)								
1	62	BT + EBRT	Prostatic urethra	0	Perineal	Yes	Gracilis muscle	Failure
2	68	BT+EBRT	Membranous and prostatic urethra	0	Perineal	No	Gracilis muscle	Success after 1 attempt
3	59	BT + EBRT	BN/LT	2	Abdomino perineal	No	Omentum	Success after 3 attempts
4	78	ARR + EBRT	BN/LT	0	Abdomino perineal	Yes	Gracilis muscle+ proctectomy	Success after 1 attempt
5	66	RRP + EBRT	BN/LT	1	Abdomino perineal	Yes	Omentum	Success after 2 attempts
6	61	HIFU + EBRT	Prostatic urethra	0	Perineal	No	None	Failure
7	71	Chemo + EBRT + TURBT	Trigone/BN	0	Perineal	Yes	None	Failure
8	67	BT + EBRT	Prostatic urethra	0	Abdomino perineal	No	Omentum	Success after 1 attempt
9	69	BT + TURP	Prostatic urethra	1	Abdomino perineal	No	None (Glubran^®^)	Failure
10	60	BT + TURP	Prostatic urethra	2	Abdomino perineal	No	None	Failure
Surgery Group (*n* = 13)								
11	73	ARR	BN/LT	0	Perineal	No	None	Success after 1 attempt
12	75	Lap RP	Giant fistula involving prostatic urethra and BN/LT	2	Abdomino perineal	No	Gracilis muscle	Success after 1 attempt
13	64	RRP	Prostatic urethra	0	Perineal	No	None	Success after 1 attempt
14	63	Lap RP	Prostatic urethra	0	Perineal	No	None	Success after 1 attempt
15	59	RRP	BN/LT	0	Perineal	No	None	Success after 1 attempt
16	75	RC + ileal neobladder	Neovesico-urethral anastomosis	0	Perineal	No	None	Success after 1 attempt
17	57	ARR	BN/LT	0	Perineal	Yes	None	Success after 1 attempt
18	65	RRP	BN	0	Perineal	No	None	Success after 1 attempt
19	76	PFUI + Lap RRP	Prostatic urethra	0	Perineal	No	None	Success after 1 attempt
20	74	RRP	Prostatic urethra	0	Perineal	No	None	Success after 1 attempt
21	68	TURP + RRP	Prostatic urethra	0	Perineal	No	None	Success after 1 attempt
22	71	Lap RRP	BN	1	Perineal	Yes	None	Success after 1 attempt
23	79	Lap RRP	Prostatic urethra	0	Perineal	No	None	Success after 1 attempt

ARR: anterior resection of rectum; BN/LT: bladder neck/ low trigonal; BT: brachytherapy; EBRT: external beam radiotherapy; HIFU: high-intensity focused ultrasound; Lap RP: laparoscopic radical prostatectomy; M: months; RC: radical cystectomy; RRP: radical retropubic prostatectomy; TURP: transurethral resection of prostate.

**Table 2:** Patients’ comorbidities and high-risk features.

	Group 1 (*n* = 10)	Group 2 (*n* = 13)	*p*-Value
Mean Age (years) (Min–Max)	66.10 (59–78)	69.15 (57–79)	0.279
Body Mass Index (kg/m^2^) (Min–Max)	26.9 (18.9–31.2)	26.29 (21.0–31.1)	0.686
Serum Albumin (g/dL) (Min–Max)	3.81 (3.1–4.6)	3.60 (2.8–4.32)	0.284
Smoking (pts/%)	4/10 (40%)	2/13 (15.4%)	0.199
Diabetes (pts/%)	2/10 (20%)	4/13 (30.8%)	0.581
Hypertension (pts/%)	5/10 (50%)	6/13 (46.2%)	0.863

**Table 3:** Surgical outcomes according to patient clinical characteristics.

	Group 1 (Non-Surgical)	Group 2 (Surgical)	Total
Surgical success after n° of attempts			
1	3	13	16/23 (70%)
2	1	0	1/23 (4%)
3	1	0	1/23 (4%)
Failures	5	0	5/23 (22%)
Total	10/23 (43%)	13/23 (57%)	
Surgical approach			
Perineal	4/10 (40%)	12/13 (92%)	16/23 (70%)
Abdominoperineal	6/10 (60%)	1/13 (8%)	7/23 (30%)
Interposition flap			
Gracilis	3	1	4/23 (17%)
Omentum	3	0	3/23 (13%)
Total	6/10 (60%)	1/13 (8%)	
Dual permanent diversion			
Required	5	0	5/23 (22%)
Not Required	5	13	18/23 (78%)
Total	5/10 (50%)	0/13 (0%)	

## Data Availability

Not applicable.

## Abbreviations

BT: brachytherapy; CT: computed tomography; DM: diabetes mellitus; FU: follow-up; HIFU: high intensity focused ultrasound; MRI: magnetic resonance imaging; QoL: quality of life; RUF: rectourethral fistula; RUG: retrograde urethrogram; VCUG: voiding cystourethrogram.
